# Mr. Adams's Case of Pins Swallowed

**Published:** 1801-03

**Authors:** William Adams

**Affiliations:** Rochester


### *To the Editors of the Medical and Phyſical Journal.*

Gentlemen,

A GIRL, fourteen years old, was ſuddenly ſeized with ſuch violent ſpaſms about the larynx, the ſides of the cheſt, and the diaphragm, that it was feared ſhe would ſoon expire. So quick was the recurrence of thoſe ſpaſms, that ſhe could neither eat nor drink. She complained of a ball aſcending from the ſtomach into the throat, and there exciting the ſpaſms.

Her breathing was difficult, ſhort, and attended with a hiſſing.

Her countenance was fluſhed.

Her

Her eyes were ſwollen; and the tunica conjunctiva was ſomewhat inflamed.

Her languor and diſinclination to move were extreme. She had an averſion to food of all ſorts, and even to drink.

Her body was rather coſtive.

She ſpat a mucus of a bluiſh green colour, which taſted, ſhe ſaid, very diſagreeably: But ſhe could not be made to compare the taſte to any other. The ſpitting was ſomewhat profuſe.

She never ſlept for many days and nights; and there was alſo a ſlight cough.

I ordered an enema, gave her Kali ſaturated with lemon juice, and the camphor mixture, with T. Fœtida, and applied a large bliſter to the pit of the ſtomach.

Theſe medicines procured no relief. I then gave pills, with camphor and opium, and applied a plaſter of opiate confection to the larynx. All ſeemed in vain, the girl grew weaker and weaker, and death ſeemed to approach by rapid ſtrides.

I then requeſted the opinion of Dr. Vaughan on ſo ſingular a caſe, whoſe attention to the poor girl merits my warmeſt thanks.

As there was once an inclination to vomit, it was encouraged; but without any good effect. To obviate coſtiveneſs, induced by the opium, the powder of ſcammony, with calomel, was given; and forty drops of tincture of opium after its operation. Every enquiry as to the riſe of the diſeaſe, was made of the girl and her friends; but for a long time nothing to direct us could be detected in their anſwers.

At length, conſidering particularly the mucus diſcharged ſo conſtantly, the girl was aſked, if ſhe could recollect having ſwallowed any thing uncommon?—To which ſhe anſwered; that ſix months ago, as nearly as ſhe could gueſs, ſhe ſwallowed two pins: But ſhe did not think them the cauſe of her complaint, as ſhe had never felt any pain or inconvenience from them.—She added further, that ſhe was certain they had paſſed from her by ſtool, as ſhe had felt them pricking the anus.

It was concluded, that the green mucus owed its green colour to the pins, and that they, or one of them, had not been voided.

A vomit was by conſequence given again; and, ſtrange to tell, the two pins were diſcharged by the mouth: Corroded indeed, but in the proper form, and only bent to almoſt a right angle.

Should the above relation of ſo ſingular a circumſtance merit a place in the Medical and Phyſical Journal, the inſertion will oblige

Your's, &c.

